# Antiretroviral treatment, management challenges and outcomes in perinatally HIV-infected adolescents

**DOI:** 10.7448/IAS.16.1.18579

**Published:** 2013-06-18

**Authors:** Allison L Agwu, Lee Fairlie

**Affiliations:** 1 Division of Paediatric Infectious Diseases, Department of Paediatrics, Johns Hopkins School of Medicine Baltimore, MD, USA; 2 Division of Infectious Diseases, Department of Medicine, Johns Hopkins School of Medicine Baltimore, MD, USA; 3 Wits Reproductive Health and HIV Institute (WRHI), University of the Witwatersrand, Johannesburg, South Africa

**Keywords:** perinatally HIV-infected, adolescents, combination antiretroviral therapy, management, resistance, outcomes

## Abstract

Three decades into the HIV/AIDS epidemic there is a growing cohort of perinatally HIV-infected adolescents globally. Their survival into adolescence and beyond represent one of the major successes in the battle against the disease that has claimed the lives of millions of children. This population is diverse and there are unique issues related to antiretroviral treatment and management. Drawing from the literature and experience, this paper discusses several broad areas related to antiretroviral management, including: 1) diverse presentation of HIV, (2) use of combination antiretroviral therapy including in the setting of co-morbidities and rapid growth and development, (3) challenges of cART, including nonadherence, resistance, and management of the highly treatment-experienced adolescent patient, (4) additional unique concerns and management issues related to PHIV-infected adolescents, including the consequences of longterm inflammation, risk of transmission, and transitions to adult care. In each section, the experience in both resource-rich and limited settings are discussed with the aim of highlighting the differences and importantly the similarities, to share lessons learnt and provide insight into the multi-faceted approaches that may be needed to address the challenges faced by this unique and resilient population.

## Introduction

With successful strategies for Prevention of Mother to Child Transmission (PMTCT), fewer infants are acquiring HIV perinatally or through breastfeeding, resulting in fewer children requiring HIV care. There are, however, approximately 2,000,000 children living with HIV globally, 90% of whom live in sub-Saharan Africa [1]. The current treatment guidelines recommend combination antiretroviral therapy (cART) initiation in infancy to prevent HIV-related morbidity and mortality [2,3]. It is expected that the majority of children who are diagnosed and treated early will survive into adolescence and adulthood [4]. Significant numbers of perinatally HIV (PHIV)-infected children newly diagnosed later in childhood only initiate cART as they approach adolescence. Knowledge of the clinical and psychosocial complexities of managing adolescent patients will be essential for both child care practitioners having their patients graduate to adolescence and adulthood, and adult care practitioners who care for adolescents as they transition to adult clinical settings [4]. Lessons learned from the decades of managing PHIV-infected adolescents in resource-rich countries will be invaluable to resource-limited countries where the burden of infection is greatest, and where cART treatment has lagged behind. To this aim, we review key differences in PHIV-infected adolescents in resource-rich vs. resource-limited settings, from diagnosis and presentation to cART recommendations and challenges, with particular emphasis on non-adherence, resistance and management strategies.

## Diagnosis and presenting features of HIV-infected adolescents

There is a wide spectrum in timing of diagnosis and entry into care for PHIV-infected adolescents. In the United States, Europe and other resource-rich settings, perinatal HIV infection has been contained by the implementation of maternal testing and PMTCT programmes since the 1990s, early testing of HIV-exposed infants, and close follow up of HIV-infected children through adolescence. In the United Kingdom and Ireland, for example, 62% of the current adolescent population presented to care at a year of age or less [5,6]. A few PHIV-infected adolescents are identified late in resource-rich settings, usually due to unknown maternal infection and missed opportunities for diagnosis [7]. Suspicion of PHIV infection should arise where there is no history of sexual activity or risk behaviours, no sexual abuse, and history of maternal risk factors, HIV diagnosis, unexplained illness or death [8,9]. High mortality rates described in PHIV-infected children under the age of two years in the pre-cART era suggest that those who survive untreated into adolescence may be slow or non-progressors [5,6,10].

In resource-limited settings, aggressive measures to improve PMTCT and infant follow-up and testing have resulted in lower transmission rates in recent years, but many PHIV-infected adolescents will not have benefited from these programmes [1,11]. A sizable number of PHIV-infected adolescents only enter care after being diagnosed during routine clinic visits, hospital admissions for illness or as part of research studies. These late presenting adolescents frequently are clinically and immunologically severely compromised, with high risk of morbidity and mortality particularly for those diagnosed in hospital settings [9,12–14]. Growth stunting and pubertal delay is common and the majority of adolescents diagnosed late have World Health Organization (WHO) Stage 3 or 4 disease, are often diagnosed with tuberculosis (TB) and may present with opportunistic infections (OIs), such as Cryptococcal disease [12–15]. Up to 75% of these PHIV-infected youth have CD4 counts below 200 cells/mm^3^ at presentation and are desperately in need of treatment [9].

## cART initiation in PHIV-infected adolescents

Essentially, most PHIV-infected adolescents that are in care have met criteria for treatment in the past or meet criteria for treatment now and should be on cART; however, there are those that are initiating cART for the first time [9–13]. In general, recommendations for cART initiation in adolescents ≥13 years of age are included in the adult guidelines for treatment and management. Both adult and paediatric guidelines alike include remarks about adolescent patients regarding dosing and management challenges, and considering regimens with a higher barrier to resistance given adherence challenges in adolescents [3,16–18]. The physiologic changes (e.g., puberty, rapid growth) that occur in adolescence result in altered pharmacokinetics. Therefore, while it is generally appropriate for post-pubertal adolescents to be dosed with cART according to adult guidelines, adolescents in early puberty should be dosed according to the paediatric guidelines which factor in dosages by weight and Tanner staging. Several of the major guidelines for cART initiation are summarized in Table 1.

**Table 1:** Guidelines for initiation of combination antiretroviral treatment in adolescents

Guidelines (date)	Clinical criteria	CD4 count absolute (cells/mm^3^)	Initial regimen	Definition of virologic failure	Second line regimen
World Health Organization [24]	WHO Stage 3 or 4 disease TB or HBV co-infection regardless of CD4 count	<350	NNRTI plus 2 NRTI's (one of which either AZT or TDF) Reduce stavudine use ABC or DDI may be used as back-up options	HIV RNA>5000 copies/mL after at least 6 months of ART	Ritonavir-boosted PI (Atazanavir or lopinavir/ritonavir preferred) and 2 NRTI's (one of which either AZT or TDF) (ABC and DDI no longer recommended)
USA Department of Health and Services [17,18]	AIDS or significant symptoms (Category C or most Category B conditions) Regardless of CD4 count Pregnancy, AIDS-defining illness, HIV-associated nephropathy (HIVAN), and HBV	Adult: all should initiate cART Strongest recommendation for CD4<350 Paediatric: ≥5: CD4<500 (asymptomatic); mildly symptomatic (CD4>500)	Preferred regimens: 2NRTIs+NNRTI/PI Adult: preferred: EFV/TDF/FTC ATV/r+TDF/FTC DRV/r+TDF/FTC RAL+TDF/FTC Paediatric: ≥6 years: ATV/r+TDF/FTC or 3TC	HIV RNA>200 copies/mL after 6 months of therapy	≥2 fully active agents from more than 1 class; guided by genotyping and prior regimens
Pediatric Network for Treatment of AIDS [16]	CDC stage B and C WHO Stage 3 and 4	<350 Consider if VL>100,000 copies/mL	ABC+3TC+EFV Consider PI^ in children/adolescents at high risk of poor adherence. HLA genotype −B*5701+ use AZT +3TC/FTC+EFV/lop/r >40 kg TDF can replace ABC	Guidelines refer to PENPACT-1 study [22]: NNRTI: switch at VL>1000 copies/mL Pi: switch if VL>30,000 copies/mL	LPV/r+AZT+TDF or LPV/r+ABC+DDI (depending on initial regimen) PI-based first line, switch to EFV with same NRTI backbone
South Africa [25]	WHO Stage 4 disease, TB co-infection Accelerate if MDR/XDR or WHO 4	<350 Accelerate if CD4<200	NVP/EFV+TDF+3TC/FTC In adolescents<40 kg or<16 years TDF is replaced by ABC GFR<50 mL/min per 1.73 m2 AZT replaces TDF	VL>1000 copies/mL consecutively 1–3 months apart	TDF/AZT (depending on initial regimen)+3TC/FTC+lop/r
Thailand [26]	AIDS-defining illness and HIV-related symptomatic Pregnant (WHO option B)	<350	NVP/EFV+AZT/TDF+3TC/FTC or Lop/r+2 NRTI's (Alternative NRTIs ABC/DDI/D4T +3TC; alternative PI's ATV/r; DRV/r: SQV/r)	VL>400 copies/mL after 6 months or>50 copies/mL after 12 months of ART DRT if VL>2000 copies/mL	Based on genotyping

^ Alternative PIs: darunavir/r, atazanavir/r, fosamprenavir/r and saquinavir/r; ABC = abacavir, AZT=zidovudine, ATV=atazanavir, D4T=stavudine, DDI=didanosine, DRV=darunavir, EFV=efavirenz, FTC=emtricitabine, Lop/r=lopinavir/ritonavir, NRTI=nucleoside reverse transcriptase inhibitor, NNRTI=non-nucleoside reverse transcriptase inhibitor, NVP=nevirapine, PI=protease inhibitor, TDF=tenofovir, /r=ritonavir boosting, 3TC=lamivudine.

## Combination ART utilization among PHIV-infected adolescents

Many PHIV-infected adolescents currently in HIV treatment programmes in sub-Saharan Africa were diagnosed in the first few years of life, starting cART at a median age between 3.6 and 4.6 years old [19–21]. Data quantifying the proportion of PHIV-infected adolescents worldwide who are eligible to receive cART and are being treated is not readily available as the WHO and other entities present data in “under 15 years” and “>=15 years” categories [11]. While the estimated number of HIV-infected children under the age of 15 years receiving cART has improved overall, there are large disparities (6–65%) in the proportion of children who need and are receiving ART, with the largest disparities documented in North Africa and the Middle East (6% [3–7%]) and West and Central Africa (9% [8–11%]) [11]. As the priority is to get the youngest children on therapy and many of the youth who are not already treated are being identified in late childhood and even in adolescence, there may even be a greater disparity in treatment for PHIV-infected adolescents. By contrast, approximately 80% of the PHIV-infected adolescents in resource-rich countries have been on longstanding cART, many having initiated therapy when they were under two years old [10,22,23].

## Challenges of cART in PHIV-infected adolescents

There are many practical considerations when initiating cART in all patients, regardless of age, including drug-drug interactions, co-morbid conditions (e.g., HBV, TB, renal and liver disease), and access and affordability [16–18,24–26]. The unique considerations and challenges to cART use in PHIV-infected adolescents, including physiologic, developmental, and psychosocial considerations, are outlined in Table 2. There are additional concerns about potential side effects, for example, bone and renal toxicity with tenofovir in the rapidly growing adolescent, which should be considered prior to cART prescription. These concerns are magnified in low weight adolescents where appropriate lower dose formulations are not available, a common problem in resource-limited countries [27]. These are discussed in other sections of this issue.

**Table 2:** Challenges of cART treatment in PHIV-infected adolescents

Problem	Implication	Solution
*Physiologic*		
Rapid growth and puberty with changes in fat and muscle mass	Altered pharmacokinetics with suboptimal drug levels	Routine dose adjustment per weight and Tanner stage assessment
Weight stunting and delayed puberty	Over-dosage of ART with potential increased toxicity risks	Routine dose adjustment per weight and Tanner stage assessment
Oro-facial motor abnormalities or lesions (e.g. candidiasis, poor dentition)	Difficulty with swallowing ART→decreased adherence	Select regimens with ART agents available in liquid or powder formulations (e.g. AZT, 3TC, ABC), or are crushable or dissolvable or allow the capsules to be opened (e.g. ATV, DRV, EFV, FTC, TDF) Note: co-formulat agents cannot be crushed
Poor palatability	Decreased adherence	Same as above; consider masking taste using soda, juice, apple sauce
*Adverse effects*		
GI intolerance (e.g. nausea, diarrhoea)	Decreased adherence	Take with meals Alter timing of administration (e.g. nighttime dosing) Anti-emetic, anti-diarrhoeal agents Consider alternative regimen
Central nervous system side effects (e.g. altered sensorium, unusual dreams, headache)	Decreased adherence	Alter timing of administration (e.g. nighttime dosing) Consider alternative regimen
Change in physical appearance (e.g. sclera icterus with ATV, facial lipoatrophy with D4T)	Decreased adherence	Consider alternative regimen
*Pharmacokinetic*		
Drug-drug interactions Rifampicin-based TB co-treatment with boosted protease inhibitor (PI) therapy Hormonal contraceptives and PI	Suboptimal PI levels Suboptimal hormonal levels with increased risk of pregnancy	Increased boosting with ritonavir or double dosing the PI For females using ritonavir-boosted PIs and combination hormonal contraceptives (pills, patches and rings) or progestin-only pills, the use of an alternative contraceptive methods or dual contraceptive methods is recommended
*Co-morbid conditions*		
Malaria, low nutritional status and advanced HIV disease	Increased risk of anaemia with certain ARVs (e.g. zidovudine)	Regular assessment of hAemoglobin levels at initiation, 1 month, 3 months and then every 6 month or symptoms
Cognitive impairment due to HIV encephalopathy, longstanding HIV infection	Difficulty in understanding HIV disease and benefits of cART → decreased adherence	Simplified regimens, cognitive age-appropriate education, high barrier to resistance regimens
*Developmental stage*		
Concrete thinking and emotional immaturity	Difficulty in understanding consequences of HIV and poor adherence → decreased adherence	Simplified regimens, cognitive age-appropriate education, high barrier to resistance regimens Address adherence frequently

ABC=abacavir, AZT=zidovudine, ATV=atazanavir, D4T=stavudine, DDI=didanosine, DRV=darunavir, EFV=efavirenz, FTC=emtricitabine, Lop/r=lopinavir/ritonavir, NRTI=nucleoside reverse transcriptase inhibitor, NNRTI=non-nucleoside reverse transcriptase inhibitor, NVP=nevirapine, PI=protease inhibitor, TDF=tenofovir, /r=ritonavir boosting, 3TC=lamivudine.

## Non-adherence to cART

A period of significant physical and psychosocial evolution [28] (e.g., concrete thinking, invincibility, risk taking, autonomy, decreased parental supervision), adolescent patients with chronic diseases such as cystic fibrosis, congenital cardiac disease, diabetes, and HIV often have decreased adherence with associated increased morbidity and mortality [6,29,30]. Successful clinical, immunological, and virological outcomes on cART are dependent on at least 95% adherence to the regimen [31]. Self-reported adherence in PHIV-infected adolescents may be anywhere between 40 and 84% in resource-rich countries [32–40], a rate lower than reported for adults. In a sub-Saharan African cohort, the numbers of adolescents achieving 100% adherence estimated by pharmacy refills, was lower than that for adults, with 20.7% at 6 months, 14.3% at 12 months, 6.6% at 24 months compared to 100% adherence in adults in 40.5%, 27.9%, and 20.6% at each time point, respectively; (*p*<0.01) [41]. Chandwani *et al*. reported that 31% of PHIV-infected adolescents were incompletely adherent in a US-based study, a rate not statistically different from non-PHIV-infected adolescents [37]. Non-adherence was associated with ever having had an AIDS diagnosis, possibly reflecting a chronic pattern of poor adherence resulting in disease progression. Additionally, older age has consistently been related to poor adherence in both resource-rich and limited countries, with adolescents above 15 years of age having a greater risk of non-adherence compared to younger adolescents [35,39,42,43].

### Adherence barriers

Non-adherence is the single most significant challenge to successful management of HIV-infected individuals, especially adolescents. It may be due to any combination of structural, patient-related, provider-related, medication-related, disease-related, and psychological barriers. Adherence is not stagnant and needs to be assessed continuously as the factors leading to non-adherence may change over time, necessitating different approaches to address them. Given the differences between PHIV-infected adolescents in resource-rich and resource-limited settings, there are likely similarities and differences between adherence barriers in those settings.

### Adherence barriers common to adolescents in both resource-rich and resource-limited settings

Lifestyle barriers such as forgetting, worrying about disclosure of HIV status, falling asleep before taking cART, being away from home, and busy and varied schedules including school attendance are common to both settings [32,33,37,38,44–46]. These factors may impact adolescents with good adherence and ways of optimizing adherence despite life's demands need to be sought [33].

Physical factors, such as behavioural and cognitive issues may further impact on adherence barriers related to lifestyle [37]. Feeling well may be associated with non-adherence by resultant complacency about cART, leading to passivity and neglecting to take ART [37].

Medication-related barriers are also common in PHIV-infected adolescents and include treatment fatigue [44,47], complexity of regimens including pill burden and dosing frequency, and palatability of cART, particularly drugs such as nelfinavir and ritonavir [32,37,38,45,48]. Where possible regimens should be simplified to fixed-dose combination tablets to improve convenience, tolerability and adherence [33,48]. However, as adolescents age and become more treatment experienced, requiring complex regimens because of poor adherence and subsequent resistance, simplified regimens become less possible, compounding the problem [37]. Adverse drug effects, from nuisance ones such as nausea and diarrhoea, to long-term toxicities such as lipodystrophy may also cause non-adherence [49,50].

Poor treatment knowledge and understanding of the benefits of taking cART as a non-curative intervention may impact adherence [45]. Also, adolescents may be emotionally unprepared for cART, particularly if they have been newly diagnosed or recently disclosed to [45]. In fact, non-disclosure of HIV status to PHIV-infected adolescents by caregivers may impact adherence, particularly when adolescents begin to question their cART regimen and express regimen fatigue [33,51]. Disclosure stressors ranked second to medication stressors in a study investigating the impact of adolescent disclosure to friends, revealing that disclosing to more than one friend was linked to less medication hiding, with an increased CD4 count and percentage, but no change in viral load [47,52].

A high percentage of PHIV-infected adolescents have experienced the loss of a primary caregiver, and parents who have survived are frequently ill, with resultant [6,19,36] depression and psychological distress which may impact adherence [30,33,53]. The coping mechanisms employed by PHIV-infected adolescents to deal with stressors are directly linked to non-adherence. Specifically, those experiencing adherence problems most commonly use withdrawal and passive emotional regulation and less commonly use problem solving or social support as coping mechanisms, possibly because of fear of stigma or unwanted disclosure. Passive coping style is also associated with depression and poorer psychological adjustment [47].

### Resource-rich settings

Psychological factors related to non-adherence are more commonly described in the literature from resource-rich locales, possibly due to under-reporting of these factors in resource-limited settings. Low self efficacy (sense of one's, ability to adhere to prescribed medication) and low outcome expectancy (ones belief in the benefits of taking a prescribed medication) are strongly associated with poor adherence in adolescents [34,46]. Mental illness as a standalone factor has not consistently been shown to affect adherence [31,34,54]. Non-adherence is associated with depression and anxiety, with those receiving antipsychotic drugs and having more than one neurologic diagnosis having improved adherence possibly due to improved observation by caregivers and healthcare providers [39]. In the LEGACY cohort of PHIV-infected US adolescents, psychiatric diagnosis which included mood disorders, Attention Deficit Hyperactivity Disorder (ADHD) and disruptive behaviour disorders was significantly associated with one of three risky health behaviours including adherence problems in 72%, preadult sexual activity in 12% and substance abuse in 9% [40].

### Resource-limited settings

Adolescents who experience structural problems such as lack of medical insurance, problems with work or school, concerns about dealing with family and looking after children, housing instability, lack of transportation to clinic visits or to obtain medications, may have lower adherence. While these issues exist commonly in resource-rich settings, they may be even more prevalent in resource-limited settings particularly those where social and political instability prevail [34,42,44,46]. Additionally, the higher prevalence of co-morbidities in resource-limited settings such as tuberculosis (drug sensitive and resistant), malaria, malnutrition, and the consequent polypharmacy and drug-drug interactions resulting from treatment may also impact adherence. Lastly, the relative lack of healthcare professionals (medical care providers, support staff, psychologists, social workers, and counsellors) experienced in adolescent healthcare management may further impact the adherence counselling and support needed for PHIV-infected adolescents in resource-limited settings.

## Interventions that improve adherence

There is no gold standard intervention to address adherence as it is a highly individualized process. Time during every encounter should be spent assessing adherence to medications [55]. Interventions need to be tailored to the individual adolescent's needs, and multiple modalities may be necessary to address non-adherence. Particularly for adolescents who have cognitive limitations as a result of longstanding HIV, with limited support, addressing adherence may be even more complex. It is critical that approaches are multi-disciplinary and appropriate for the patient's cognitive age and psychosocial stage, given the variability that can occur with PHIV-infected adolescents [32]. Additionally, it is important for the provider to not become frustrated with the patient as multiple failures may precede successful improvements in adherence; and addressing adherence improvement is ongoing as non-adherence can recur. When possible, involving the parent/caregiver in addressing non-adherence may be critical as there are often discrepancies in perception of adherence between the parent/caregiver and the adolescent as the responsibility for medication taking is transferred to the adolescent. Interventions involving both parties are crucial to improving adherence [32]. Some strategies to improve adherence are outlined in Table 3.

**Table 3:** Strategies to address non-adherence in perinatally HIV-infected adolescents

Strategy
Medication-related barriers Reduced pill burden (e.g. once daily/fixed-dose combinations) Palatable formulations (liquid, powder, crushing) Management of side effects Anti-nausea, anti-diarrhoeal agents Change timing of dosing (e.g. nighttime dosing) Regimen change
Patient-related factors Disclosure Counselling to deal with loss/trauma Treatment of concurrent psych diagnosis (e.g. anxiety, depression, substance abuse) Education about HIV and benefits of Cart
Behavioural interventions Motivational interviewing Counselling, support groups Life skills education with time-management and prioritization Parental/caregiver involvement Buddy systems Adherence clubs Peer motivators/educators Activity triggers (e.g. meals) Calendars Technological interventions (e.g. cell phone (calls or SMS texts, watches, beepers)) Pill boxes Pharmacy clinic Directly observed therapy
Structural barriers Address barriers such as transportation, insurance, child care, clinic hours Education of clinic staff about cognitive and development stage of adolescence

## Treatment outcomes in PHIV-infected adolescents

In their second decade of HIV infection, the delicate balance of the virus and host is altered and PHIV-infected adolescents, in the absence of effective cART, will usually have immunologic deterioration, with development of clinical illness, including OIs [8,12]. Studies have shown that adolescents, particularly older adolescents, comprise the majority of PHIV-infected children being hospitalized and have highest rates of morbidity and mortality [56]. Many PHIV-infected adolescents in longitudinal cohorts, mostly from well-resourced countries, remain stable on cART with good adherence, retention in care and have good clinical, immunological, and virological outcomes [6,10,22,23]. Despite these successes, adolescence is a high risk period for adherence problems, with clinical, immunological, and virological outcomes determined by adherence to ART, associated disease progression and availability of new cART regimens in those experiencing first, second or third line ART failure.

## Clinical outcomes in PHIV-infected adolescents

Data describing longitudinal follow up in PHIV-infected adolescents from resource-rich settings show that up to 26% had ever had a clinical Centers of Disease Control and Prevention (CDC) C disease classification, indicating severe clinical immunocompromise during their lifetime [5,6,10,22]. Despite this, 85% of adolescents were well at recent follow up and weight, height, and body mass index (BMI) was well maintained, consistent with population norms [6,10]. Studies from the UK, Ireland, and the US have shown reduction in mortality of up to 76% between 1996–2006 in children and adolescents on cART and significant reduction in hospital admission rates over the same period in the Collaborative HIV Paediatric Study (CHIPS) cohort [9,10,22,57]. Mortality outcomes from sub-Saharan Africa suggest no difference in mortality between adults and adolescents. In a comparative study from South Africa, mortality rate was 2.9 per 100 person-years [95% confidence interval (CI) 2.3–3.7], with no differences between adolescents (9–19 years) and young adults (20–28 years), with similar findings in a Ugandan study, where adolescents (11–19 years) and adults had higher mortality rates (8.5 and 10% respectively) compared to children<10 years (5.4%), but no differences between them [58,59].

## Immunological outcomes of cART in PHIV-infected adolescents

Immunologic characteristics of PHIV-infected youth in care show robust CD4 counts in both resource-rich and limited settings. The median CD4% for PHIV-infected youth, entering the adolescent master protocol cohort (median age of 12.2 years) in the United States was 33% [35]. In the CHIPS and French (median age 15 years) cohorts CD4 at last follow up was 554 cells/mm^3^ [IQR 324–802] and 550 cells/mm^3^ [IQR 832–861], respectively [6,10,22]. In a Zimbabwean cohort (mean age 14 years) CD4 count was 384 cells/mm^3^ [12]. Younger adults (18–30 years) have better immune recovery than older adults (>30 years), related to high thymic scores and immune restoration driven by therapy-associated reversal of immune reactivation giving them greater capacity to recruit and repopulate CD4 cells [60]. This can be extrapolated to adolescents, who are newly initiating ART, where high initial increases in CD4 percentage in the first year of cART initiation are sustained for five years of follow-up [57]. In a report from South Africa, adolescents had a greater change in median CD4 from baseline to 48 weeks (373 vs. 187 cells/µL; *p*=0.0001) compared to young adults (20–28 years) in both the non-PHIV- and PHIV-infected groups [58].

## Virological outcomes in PHIV-infected adolescents

Assuming that there is no underlying resistance to a regimen that is selected, virologic suppression for adolescents should be similar to adults starting on similar regimens. However, virologic suppression rates in longitudinal adolescent cohorts are lower than those in adults, ranging from 28 to 78% compared to as high as 90% for adults on similar regimens [6,10,11,30,35,36,38,39,58,61,62]. In one study, the rates of virological failure (defined as initially achieving virological suppression with two subsequent viral loads >400 copies/mL) were significantly higher in adolescents compared to young adults 8.2 (95% CI 4.6–14.4) and 5.0 (95% CI 4.1–6.1) per 100 person-years, respectively (*p*=0.001). This association was weakened in a sub-analysis comparing PHIV-infected adolescents to young adults [AHR 1.51 (95% CI 0.68–3.33; *p*=0.31)] [42]. Also, a study from nine sub-Saharan African countries showed that adolescents were 70–75% less likely to have undetectable viral loads at 12, 18, and 24 months on highly active antiretroviral therapy (HAART). Adolescents who were virally suppressed at 12 months were more likely to experience viral rebound compared to adults [40].

In general, with appropriate cART, virologic suppression and a CD4 increase of 150 cells/mm^3^ should occur by six months after initiation, with the caveat that with significantly elevated viral loads and markedly suppressed CD4 counts, this may vary [18]. In settings where there is virologic or immunologic failure, underlying reasons need to be assessed in order to determine a course of action (Table 4). Upon failure of the first cART regimen, patients are then categorized as treatment-experienced, with added challenges to devising suppressive regimens and maximizing outcomes.

**Table 4:** Assessment and management of treatment failure in perinatally HIV-infected adolescents

	Virologic failure	Immunologic failure
Definition	Variable per guidelines (see Table 1)	• Failure to achieve and maintain an adequate CD4 response despite virologic suppression • Failure to improve CD4>350 cells/mm^3^ Note: Increases in CD4 counts in ARV-naive patients with initial ARV regimens are approximately 150 cells/mm^3^ over the first year
Potential causes	Patient characteristics associated with virologic failure Higher pretreatment or baseline HIV RNA level Lower pretreatment or nadir CD4 T-cell count Prior AIDS diagnosis Incomplete treatment of opportunistic infections Comorbidities (e.g. active substance abuse, depression) Presence of drug-resistant virus, either transmitted or acquired Prior treatment failure Incomplete medication adherence and missed clinic appointments ARV regimen characteristics Drug side effects and toxicities Suboptimal pharmacokinetics (variable absorption, metabolism, or, theoretically, penetration into reservoirs) Food/fasting requirements Adverse drug-drug interactions with concomitant medications Adverse drug-drug interactions with concomitant medications Suboptimal virologic potency Prescription errors Provider characteristics, such as experience in treating HIV disease Other or unknown reasons Lab error	CD4 count <200/mm^3^ when starting cART Older age Co-infection (e.g. TB, hepatitis C virus, HIV-2, human T-cell leukemia virus type 1 [HTLV-1], HTLV-2) Medications, both ARVs (e.g. ZDV TDF+didanosine [ddI]) and other medications Persistent immune activation Loss of regenerative potential of the immune system Other medical conditions
Evaluation	Confirm virologic failure by repeating HIV RNA after 1–3 months Assess for HIV-related clinical events Review ARV treatment history and response Compile and review resistance test results through tools such as the Stanford Database • Obtain new genotype while still on current cART Review medication taking behaviour and adherence, including adherence to dosing and food requirements Review concomitant meds (prescribed and over the counter and homeopathic) for drug-drug interactions Assess co-morbidities	Confirm virologic failure by repeating Assess for HIV-related clinical events Assess virologic response Review medication taking behaviour and adherence, including adherence to dosing and food requirements Review concomitant meds (prescribed and over the counter and homeopathic) for drug-drug interactions and effect on immune system Assess co-morbidities (malignancy other infections)
Interpretation	If continued virologic failure and no evolution of resistance, adherence most likely	If all investigation unremarkable, isolated immunologic failure
Management	Drug-drug interaction: resolve by discontinuing, changing the offending drug or if not possible, consider changing the ART regimen Resistance: select new regimen with at least 2 new active agents (see Table 1) Adherence: re-enforce adherence, utilize strategies (see Table 2)	If>200 cells/mm^3^ close monitoring, unclear if should prompt change in therapy, therefore not recommended

## Treatment-experienced patients

In resource-rich settings, many PHIV-infected adolescents are significantly treatment-experienced, with over a decade of antiretroviral exposure on average, often with suboptimal single and dual ART regimens before transitioning to cART when it became available [10,22]. For example, in the PHACS cohort, only 10–20% of the adolescents had cART as their first regimen, and the median number of ART agents they had been exposed to was seven [22]. Many PHIV-infected adolescents have long histories of suboptimal drug regimens, reduced drug levels due to poor absorption, drug-drug interactions, and non-adherence, which has implications for their likelihood of virologic failure and resistance, and dramatically compromises the ability to design suppressive regimens. In resource-limited settings PHIV-infected adolescents present later in childhood and usually have sensitive virus as prior exposure to suboptimal mono- or dual ART exposure is uncommon. However with increasing uptake of PMTCT and earlier cART initiation for infants and children, issues of drug-drug interactions, medication security and non-adherence, similar challenges of treatment experience seen in resource-rich settings are becoming more common [63].

### Resistance outcomes for PHIV-infected adolescents

**Figure 1:**
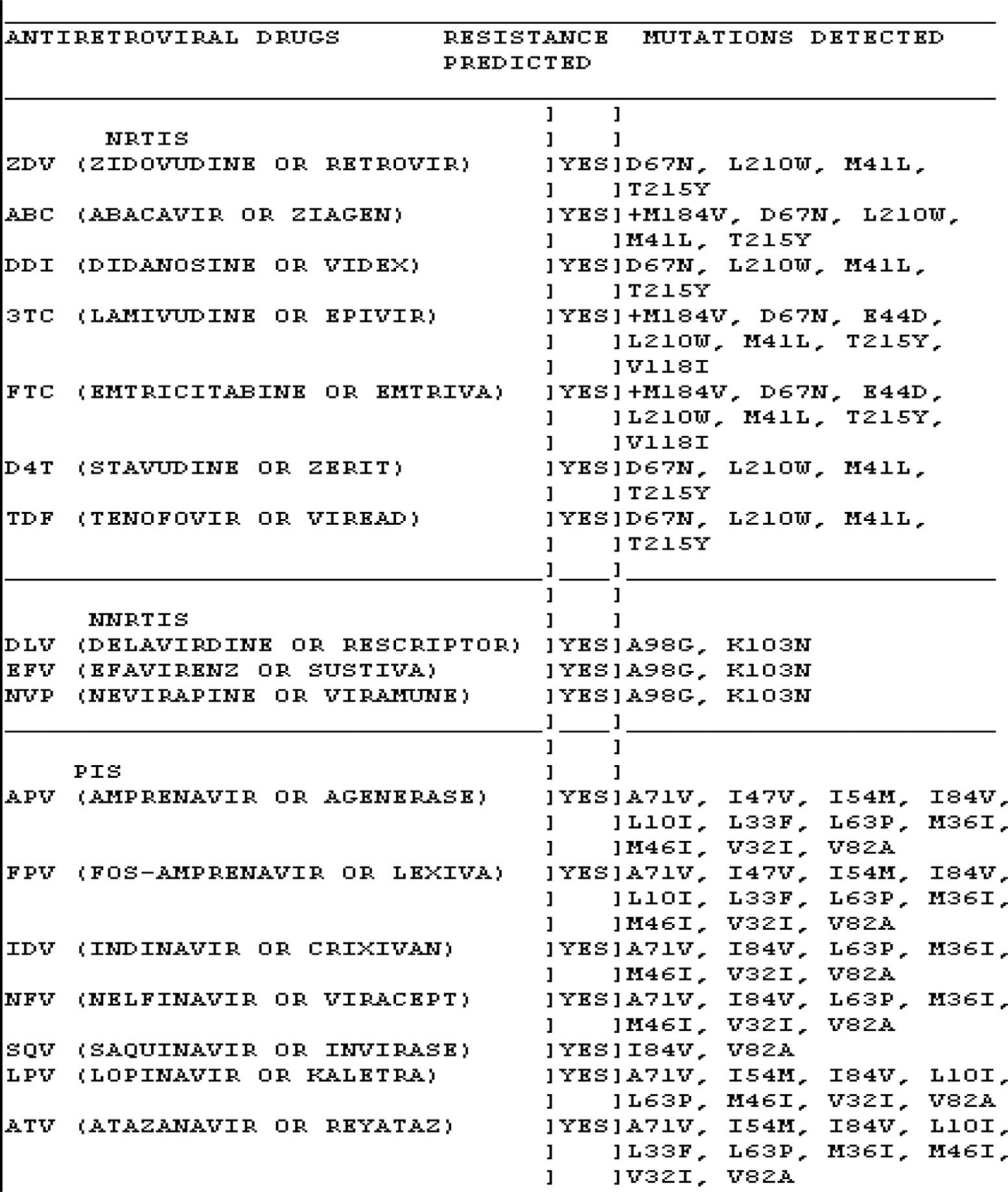
Multi-drug genotypic resistance from a treatment-experienced PHIV-infected adolescent.

In the setting of suboptimal ART drug levels there is resultant viral evolution and development of resistance [64]. Certain regimens have been associated with higher rates of resistance, e.g., prolonged failure on an NNRTI-based regimen, triple nucleoside regimens, use of ritonavir as a single PI and boosted PI regimens without additional ritonavir boosting for rifampicin-based tuberculosis co-treatment [62,65–67]. Foster *et al*. reported that in a UK and Ireland cohort, 52% of PHIV-adolescents had dual and 12% had triple-class ART resistance [6]. Genotypic resistance testing revealed NNRTI-associated mutations (i.e. 103N, 181C/I, and 190A) (65%), the NRTI mutation, M184I/V (49%), non-M184I/V NRTI mutations (thymidine analogue mutations) (57%), and major PI mutations (26%) [6]. Although not routinely assessed in most non-research resource-limited settings, when studies have assessed resistance levels, in children (not specifically adolescents) failing first and second-line regimens, 34–99% had evidence of resistance, primarily consisting of NNRTI resistance and the NRTI mutation M184V, leaving limited treatment options available in those settings (see Figure 1) [68–71]. Data on resistance in resource-limited settings are limited although being garnered by the WHO HIVResNet, the Global HIV Drug Resistance Surveillance Network, a collaboration between WHO and the International AIDS Society. The network develops standards for detecting resistance; identifies factors leading to resistance; builds and maintains monitoring capacity in developing countries through technology transfer, training and technical assistance; monitors resistance in untreated patients and samples of selected treated patients; then disseminates data in order to inform containment strategies [72]. These data are critically needed as cART uptake increases.

Emergence of drug resistance has been highly correlated with all-cause mortality, with resistance to particular classes of agents, NNRTI specifically, having a threefold higher correlation with mortality, likely due to virulence of these viral variants [73]. This correlation of resistance to morbidity and mortality has been consistently shown in several studies in various settings, resource-rich and resource-limited [74]. In analyses of paediatric cohorts, adolescents had the highest hospitalization and mortality rates, without the significant declines seen in other age groups [56].

### Strategies to help guide targeted resistance testing for adolescents in resource-limited settings

In resource-rich settings, resistance testing (genotypic and when necessary phenotypic assessment) is readily available and prudent in the setting of virologic failure in order to guide decisions about treatment. Particularly critical for adolescents who are highly treatment-experienced, is the cumulative genotype of all mutations previously documented as with decreased drug-selective pressure with poor adherence or switch to a different drug regimen, viral variants harbouring resistance may fade from the circulating plasma viral pool, but still be present in the latent reservoir, emerging when the drug-selective pressure is resumed. Also, all prior ART regimens and responses to those regimens should be reviewed to assess likelihood of residual activity and predicted presence of resistance [17,18]. In resource-limited settings, genotyping is not readily available and is often restricted for use in adolescents failing second line regimens.

### ART strategies in highly treatment-experienced PHIV-infected adolescents

Although the availability of newer agents, including integrase inhibitors and CCR5 antagonists in certain settings, has generated renewed hope for virologic suppression and immune recovery for heavily treatment-experienced PHIV-infected adolescents with extensive resistance, selecting an optimal background regimen to achieve virologic suppression is a challenge. As many PHIV-infected adolescents continue to struggle with adherence, timing of introducing the salvage regimen is critical to prevent failure on what is often the last available suppressive regimen. In a retrospective study by Wong *et al*., PHIV-infected youth on cART were assessed and their regimens characterized as optimal vs. sub-optimal based on cumulative genotypes and anticipated drug activity at the start of the regimen. More than half of the patients from each cohort had poor adherence. By 48 weeks, those in the optimal group had a greater median CD4 increase, +62 (+25 to +200) than those in the suboptimal group +8 (−93 to +54) cells/mm^3^ (*p*=0.04), and were four times more likely to have an increased CD4>50 cells/mm^3^, a difference that persisted throughout the study period. There were no differences in clinical events or accumulation of new resistance mutations between the two groups. The authors’ caution that the group of highly treatment-experienced adolescents with ongoing poor adherence could develop resistance to the optimal regimen and conclude that the choice of initiating a new regimen needs to consider adherence, adverse effects, pill burden and fear of accumulating resistance [49]. In general, the principles that guide managing treatment-experienced PHIV-infected adolescents include: switch only once adherence issues resolved, never only switch one drug in a failing regimen and do not continue therapy with a failing NNRTI regimen for prolonged periods as there is an increased risk of accumulating NNRTI resistance mutations compromising the class [75]. The approach to managing treatment failure depends on what tools are available to providers (Tables 1 and 4) and must take into consideration adherence and disease stage [76].

Providers that care for PHIV-infected adolescents have been forced to be creative in managing treatment failure in this population. Possible strategies include: bridging strategies (minimalist strategies; 3TC monotherapy; simplification strategies), and treatment de-intensification or even discontinuation [77–81]. Once treatment is initiated (person meets criteria for treatment) discontinuation has potential immunologic, virologic and inflammatory consequences and is therefore not recommended by the guidelines [17,18,63,82]. However, treatment interruption (patient or provider-initiated), so-called drug holiday, is a strategy that has been utilized to manage some PHIV-infected adolescents who are unable to adhere despite all adherence interventions, underscoring the management challenges. In the CHIPS cohort, at last follow up, 18% of PHIV-infected adolescents who were receiving cART previously, were not receiving it [6]. Similarly in a longitudinal French cohort, 16% of had discontinued therapy [10]. Siberry *et al*. examined treatment interruptions in PHACS and reported that 23% of the cohort, significantly more in the earlier birth cohort (1991–1993) vs. younger cohorts, had discontinued ART for at least one period of ≥3 months after continuous ART for ≥6 months [76]. While immunologic decline occurred overall, significant variability was seen across the cohort. In general, these alternative management strategies have proven to be safe; however, their use should be accompanied by continued adherence strengthening, close monitoring and research to determine their effectiveness. PHIV-infected adolescents may be ideal candidates for future innovative strategies such as therapeutic vaccines and novel approaches, such as depot ART should they become available.

## Additional concerns and management issues related to PHIV-infected adolescents

### Unchecked inflammation

Inflammation is increasingly being recognized as a significant consequence of HIV infection. Initially shown in adult studies, subsequent paediatric studies have also shown that PHIV-infected children have a high degree of inflammation related to uncontrolled HIV replication [83]. The sequelae of this heightened inflammation includes vascular anomalies with resultant heart disease, strokes, altered glucose metabolism, malignancy, neurologic disease, etc [84]. This inflammation is lowered, but not aborted/terminated by ART. In the PHACS cohort, markers of inflammation, coagulant, endothelial and metabolic dysfunction were assessed and correlated with ART and viremia [83]. HIV-infected children with a median age of 12.3 years had higher levels of cholesterol and triglycerides, despite lower body mass index, waist and hip circumference and percentage body fat. This cohort also had higher measurements of all of the inflammatory markers measured. Specifically, increased HIV viral load was associated with markers of inflammation and endothelial dysfunction [83]. Given that HIV infection is lifelong, and with ART there is increased survival of PHIV-infected adolescents, the sequelae of this unchecked inflammation, particularly in those non-adherent to ART, is of concern.

### Transmission

Studies have reported mixed findings regarding sexual activity in PHIV-infected adolescents with some studies reporting delayed penetrative sex in young HIV-infected adolescents [35,85] with no association between HIV status and sexual risk behaviour, and others reporting increased risk-taking behaviour, including sexual behaviour, substance abuse, and an increased risk of pregnancy [40,85–87]. A recent study of PHIV-infected adolescents revealed that 28% reported sexual intercourse with a median age of coitarche of 14 years; 62% reported unprotected sexual intercourse, and only 33% of youth disclosed their HIV status to their partners. Interestingly, of youth who did not report being sexually active at baseline, ART non-adherence was associated with sexual debut during the follow-up period. The investigators also examined genotypic resistance in the 42% of the sexually active youth that had viral loads ≥5000 copies/mL, identifying 62, 57, 38, and 22% to NRTIs, NNRTIs, PIs, and all three ARV classes, respectively. The sequelae of these unprotected acts include sexually transmitted infections and pregnancies, which have been reported in PHIV-infected adolescents. The rates of reported sexual activity and failure to use barrier protection raise concern for secondary transmission, horizontal and vertical. In the setting of non-adherence, the concern is heightened as there is a risk of transmission of resistant virus, limiting treatment options for the individual acquiring primary infection. The authors rightfully conclude that the combination of unprotected sexual activity, non-disclosure and ART resistance places partners at risk for HIV infection and call for interventions to facilitate youth adherence, safer-sex practices and disclosure [88].

## Transition to adult care

PHIV-infected adolescents often have complex psychosocial situations and clinical histories, including complicated resistance patterns [6,10]. These patients may be seen in paediatric or adult clinics where there is significant variability including, but not limited to the clinic appearance, services provided, target populations, provider–patient ratios, availability of youth-friendly services, training and experience of the clinic personnel in adolescent health and development and HIV outcomes for this population [89]. The transfer of care from a paediatric/adolescent to an adult clinic may be accompanied by significant anxiety and may lead to a disruption in care [90]. As adolescents transition between paediatric and adult clinical venues, it will be critical for providers on both sides to have a thorough understanding of the multi-faceted issues including complicated treatment histories, complex psychosocial dynamics and developmental stage, in order to effectively manage PHIV-infected adolescents and optimize outcomes after transfer.

## Gender considerations in PHIV-infected youth

In published studies of PHIV-infected adolescents, there is usually equal gender distribution between male and female PHIV-infected adolescents, a characteristic which distinguishes PHIV and non-PHIV-infected adolescents, where there tend to be varying gender distributions depending on the epidemic (e.g., majority males infected via MSM activity in the United States, and females infected through heterosexual sex in Sub-Saharan Africa). Gender may significantly affect outcomes and clinical practice in PHIV-infected adolescents for a number of reasons. Contrasting findings regarding the impact of gender on adherence and virological suppression warrant further investigation. A French cohort demonstrated greater virological suppression rates in female adolescents in a multivariate analysis of the cohort [10], while two studies in the United States reported that male gender was associated with improved adherence and virological suppression [22,39]. One study has reported lower efficacy of lopinavir in male adolescents over 12 years of age, and although the numbers in this group were insufficient to analyze statistically, the clinical and virological significance of this finding warrants further investigation [91]. Female PHIV-infected adolescents may enter puberty earlier than males which may affect safety and dosing of ARVs such as tenofovir. Use of hormonal contraceptives, particularly the combined oral contraceptive pill with concurrent PI use and the subsequent drug-drug interactions may result in reduced contraceptive efficacy with possible pregnancy in female PHIV-infected adolescents. For females using ritonavir-boosted PIs and combination hormonal contraceptives (pills, patches and rings) or progestin-only pills, the use of an alternative contraceptive method (e.g. intrauterine device [IUD]) and/or dual contraception methods is recommended (Table 2). Hormonal contraception particularly the injectable methods may result in increased HIV transmission to HIV negative partners, likely due to a combination of decreased condom use and increased vaginal inflammation and intravaginal viral load [92]. Discussion of contraceptive needs with sexually active adolescents is an important component of HIV care. Practitioners managing PHIV-infected adolescents need to be aware of these potential differences related to gender in order to provide comprehensive, safe care for this population.

## Conclusions

In conclusion, the growing cohort of PHIV-infected children that are emerging into adolescence and young adulthood require cART treatment to control viral replication, prevent immune deterioration and avert secondary transmission. Successful treatment is complicated by developmental, cognitive and psychosocial challenges that can compromise adherence leading to the development of resistance and reduced treatment options, with resultant morbidity and mortality. While recent data in adults has estimated that the life expectancy for HIV-infected individuals has improved to near normal, with significant proportions of PHIV-infected adolescents emerging into adulthood with resistant virus, continued non-adherence, and the limited pipeline of new agents, there is concern that the survival seen in HIV-infected adults may not be duplicated for PHIV-infected adolescents. Resource-rich settings are over a decade ahead of resource-limited settings in their treatment of PHIV-infected adolescents, providing foreshadowing for some of the challenges ahead for resource-limited settings and insight into the multi-faceted approaches that may be needed to address these challenges. Lessons learnt from resource-rich settings and research about the unique barriers that may exist in resource-limited settings will be critical to assuring that PHIV-infected youth continue to benefit from treatment as they navigate the challenging period of adolescence.
